# High-Level Expression of Wild-Type p53 in Melanoma Cells is Frequently Associated with Inactivity in p53 Reporter Gene Assays

**DOI:** 10.1371/journal.pone.0022096

**Published:** 2011-07-08

**Authors:** Roland Houben, Sonja Hesbacher, Corinna P. Schmid, Claudia S. Kauczok, Ulrike Flohr, Sebastian Haferkamp, Cornelia S. L. Müller, David Schrama, Jörg Wischhusen, Jürgen C. Becker

**Affiliations:** 1 Department of Dermatology, University Hospital Würzburg, Würzburg, Germany; 2 Department for Obstetrics and Gynecology, University of Würzburg, Würzburg, Germany; 3 Department of Dermatology, University of Saarland, Homburg/Saar, Germany; 4 Department of General Dermatology, Medical University of Graz, Graz, Austria; The University of Queensland, Australia

## Abstract

**Background:**

Inactivation of the p53 pathway that controls cell cycle progression, apoptosis and senescence, has been proposed to occur in virtually all human tumors and p53 is the protein most frequently mutated in human cancer. However, the mutational status of p53 in melanoma is still controversial; to clarify this notion we analysed the largest series of melanoma samples reported to date.

**Methodology/Principal Findings:**

Immunohistochemical analysis of more than 180 melanoma specimens demonstrated that high levels of p53 are expressed in the vast majority of cases. Subsequent sequencing of the p53 exons 5–8, however, revealed only in one case the presence of a mutation. Nevertheless, by means of two different p53 reporter constructs we demonstrate transcriptional inactivity of wild type p53 in 6 out of 10 melanoma cell lines; the 4 other p53 wild type melanoma cell lines exhibit p53 reporter gene activity, which can be blocked by shRNA knock down of p53.

**Conclusions/Significance:**

In melanomas expressing high levels of wild type p53 this tumor suppressor is frequently inactivated at transcriptional level.

## Introduction

p53, the “guardian of the genome”, is a transcription factor that can bind to promoter regions of hundreds of genes where it either activates or suppresses gene expression [1]. Thereby, p53 serves as a tumor suppressor by inducing cell cycle arrest, apoptosis, senescence and DNA repair [2]. In normal cells, p53 is frequently undetectable due to fast ubiquitination by mdm-2 and subsequent proteasomal degradation [3]. However, upon DNA damage and several other stresses, including oncogenic stress, the amount of p53 is increased due to disruption of its degradation [4], [5]. Notably, inactivation of p53 is one of the characteristics of cancer. Indeed, p53 has a wide spectrum of mutation types and p53 is found mutated in approximately half of all tumors [6]. Most of these aberrations are missense mutations in the central DNA binding domain disrupting the transcriptional capability and conferring different degrees of dominance over co-expressed wild-type p53 [7]. Alternatively, impairment of the p53 response to oncogenic stress has been reported which is mediated via increased degradation by viral proteins or mdm-2 overexpression [8], [9]. Moreover, p53 induction may be affected for example by loss of p14^ARF^ [10]. On a post-translational level, the transcriptionally activity of p53 can also be impaired by sequestration in the cytosol [11], by competition for DNA-binding sites by ΔNp73 [12] or functional antagonism with iASPP [13].

Recently, p53 has been suggested to be a major player suppressing progression from nevi to melanoma [14]. Studies on the frequency of p53 mutations in melanoma, however, reported relatively low but quite divergent numbers ranging from 0 to 30% [15]–[20]. To address this controversy we here evaluated a large series of more than 180 melanoma samples for p53 protein expression and genetic p53 mutations. Although p53 is generally expressed in melanoma tissues it harbours only very rarely mutations. In contrast, in 4 of 14 melanoma cell lines p53 was found to be mutant. Notably, in melanoma cell lines which have preserved the p53 wild-type geno/phenotype observed *in situ*, we demonstrate transcriptional inactivity of the protein in 6 out of 10 cell lines suggesting active silencing as the mode of p53 inactivation in melanoma.

## Results and Discussion

### Expression of p53 in melanoma

**Figure 1:**
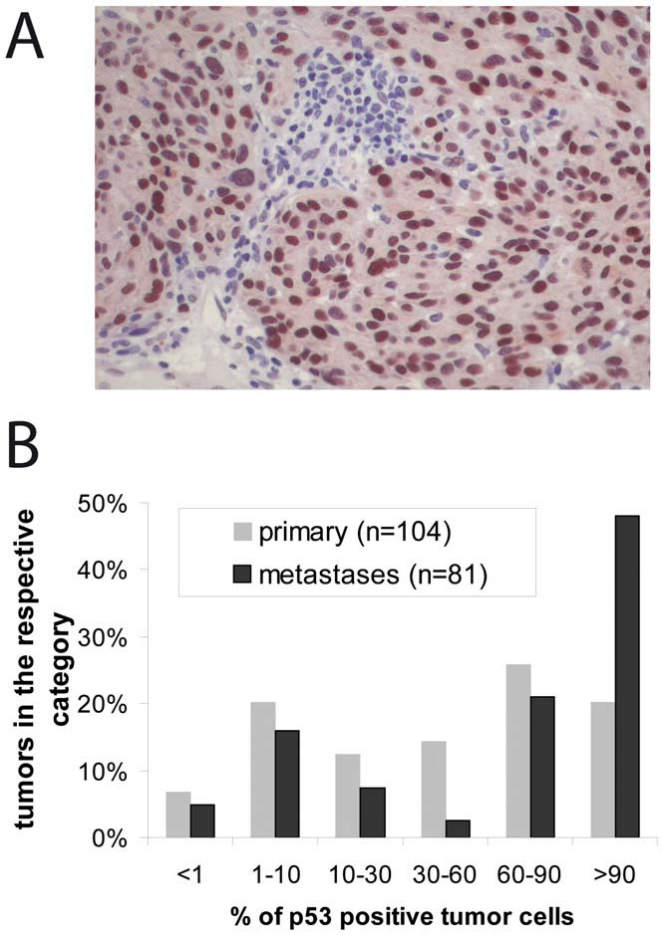
p53 expression in melanoma tissues. (**A**) Immunohistochemical detection of p53 in a primary melanoma. (**B**) 104 primary cutaneous and 81 metastatic melanoma samples were stained for p53. The percentage of positively stained tumor cells is given as bar graph.

Many tumors accumulate mutant p53 protein which is explained by a lack of p53-dependent Mdm-2 induction leading to decreased proteasomal degradation [3]. Immunohistochemical analysis of 185 melanoma samples demonstrated that in the majority of tumors high levels of p53 are expressed in a considerable proportion of the tumor cells (Figure 1 A and B). Moreover, we observed an increase in p53 expression from primary to metastatic tumors (Figure 1B), thereby confirming previous reports [21].

### Absence of p53 mutation in melanoma

**Figure 2:**
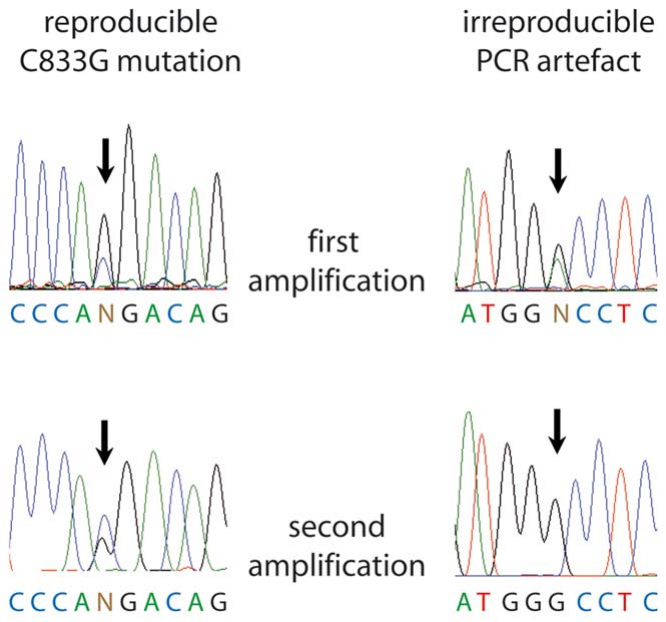
Reproducibility of p53 sequencing. Sequencing chromatograms depicting the only reproducible p53 mutation and an example of an apparent mutation which could not be reproduced in an independent PCR amplification from the same sample.

p53 mutation frequencies of 21, 24 or 29% have been described for melanoma tissues and short term melanoma cell cultures [17]–[19]. Prompted by these reports and the finding that p53 was highly expressed *in situ*, we started to analyze genomic DNA from a series of 206 paraffin-embedded melanoma tissues by nested PCR amplification and direct sequencing of the p53 exons 5–8; these exons harbour 95% of the known p53 mutations [10]. All four, three, two or only one exon were successfully amplified for 128, 34, 32 or 12 samples, respectively. In 26% of the samples, we observed irreproducible PCR artefacts which pretended possible mutations (Figure 2). However, a reproducible amino acid substitution (P278R; C833G; heterozygous; GenBank accession number: 573759) was only observed in one case (Figure 2). We do not know whether sequencing results in studies observing higher p53 mutation rates were controlled for reproducibility but reported high frequencies of silent mutations as well as double and triple mutations in one exon may be indicative of false positives [18], [19]. Furthermore, formalin-fixed paraffin-embedded material as used in these studies (like in ours) is notorious for generating PCR artefacts [22]. Based on carefully reproduced sequencing results in this - to the best of our knowledge - largest series studied to date, we now confirm previous reports describing a virtual absence of p53 mutations in melanoma tissues [15], [16], [20]. However, we have to admit that there was a considerable fraction of 78 samples for which we failed to amplify all 4 exons. Thus, we do not know the complete sequence of the p53 mutation hot spot region in this fraction of specimens. All primer binding sites, however, were located in exon-flanking introns, thus, failure of amplification does not indicate an altered coding sequence. Moreover, as we could detect p53 expression in virtually all corresponding tissues, inability of PCR amplification cannot be due to loss of both p53 alleles. Most likely, failure of amplification, is simply due to limited quality of the gDNA extracted from the formalin-fixed and paraffin-embedded tissues. This is further substantiated by the fact that for many of the respective DNAs not only amplification of more than one p53 exon but also amplification from other genes like BRAF and NRAS failed.

### p53 mutations in melanoma cell lines

**Figure 3:**
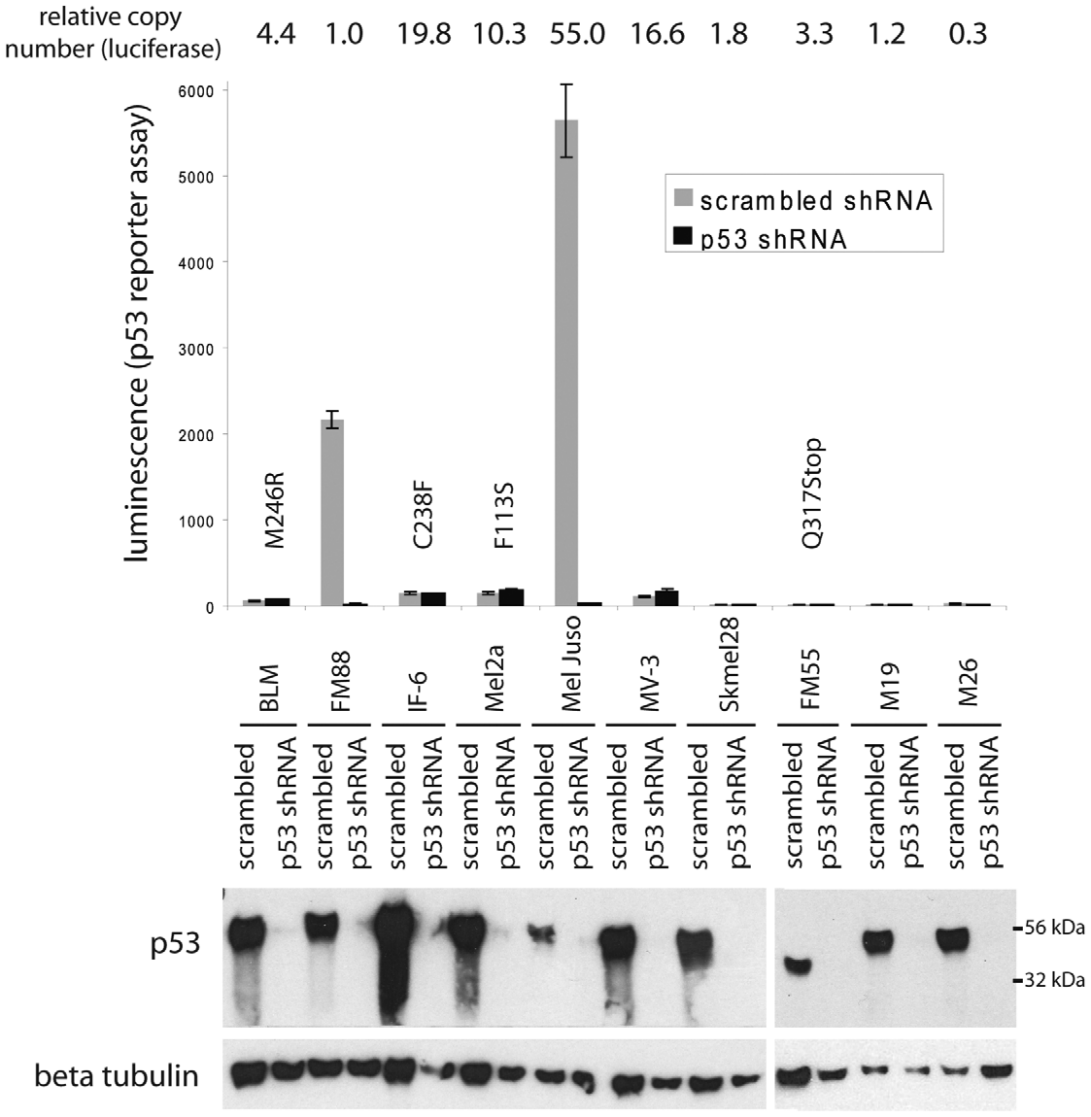
Functional inactivity in 4 out of 6 melanoma cell lines expressing wild type p53 measured by the p53 reporter construct PathDetect®. The p53 reporter construct PathDetect® encoding luciferase under the control of a p53 responsive element (14× TGCCTGGACTTGCCTGG) was transfected into the indicated melanoma cell lines. Following selection for a co-transfected puromycin resistence integration of the luciferase gene was confirmed by real time PCR and the relative copy numbers are indicated (top row). The cells were then infected with lentiviral supernatants carrying the shRNA expression construct KH1 containing either a scrambled or a sequence targeting p53 [41]. On Day 4 following infection the cells were harvested and lysates were subjected to a luciferase assay (upper part) or were analysed for expression of the indicated proteins by western blot (lower part). Sequence analysis of all p53 coding exons revealed that four cell lines carried the indicated p53 amino acid substitutions.

Notably, several of the previous studies on p53 mutations were carried out in melanoma cell lines. Thus, we analysed the mutational status of p53 in 14 melanoma cell lines by sequencing all coding exons of p53. 4 of the 14 cell lines, i.e. BLM, IF-6, Mel2a and FM55, harboured p53 mutations (Figure 3; GenBank accession numbers 573755–573758). All of the mutations have been described to be inactivating (http://www-p53.iarc.fr). Interestingly, all four sequences lacked the corresponding wild type signal indicating either homozygous mutation or more probably loss of heterozygosity at the p53 locus. In line with this, FM55 cells, where the mutation leads to a premature stop codon, express only the truncated p53 protein indicated by increased mobility in gel electrophoresis (Figure 3). Hence under culture conditions melanoma cells tend to acquire inactivating p53 mutations although *in situ* mutant p53 is not favoured [15].

### Absence of p53 reporter gene activity in 6 out of 10 wt p53 melanoma cell lines

**Figure 4:**
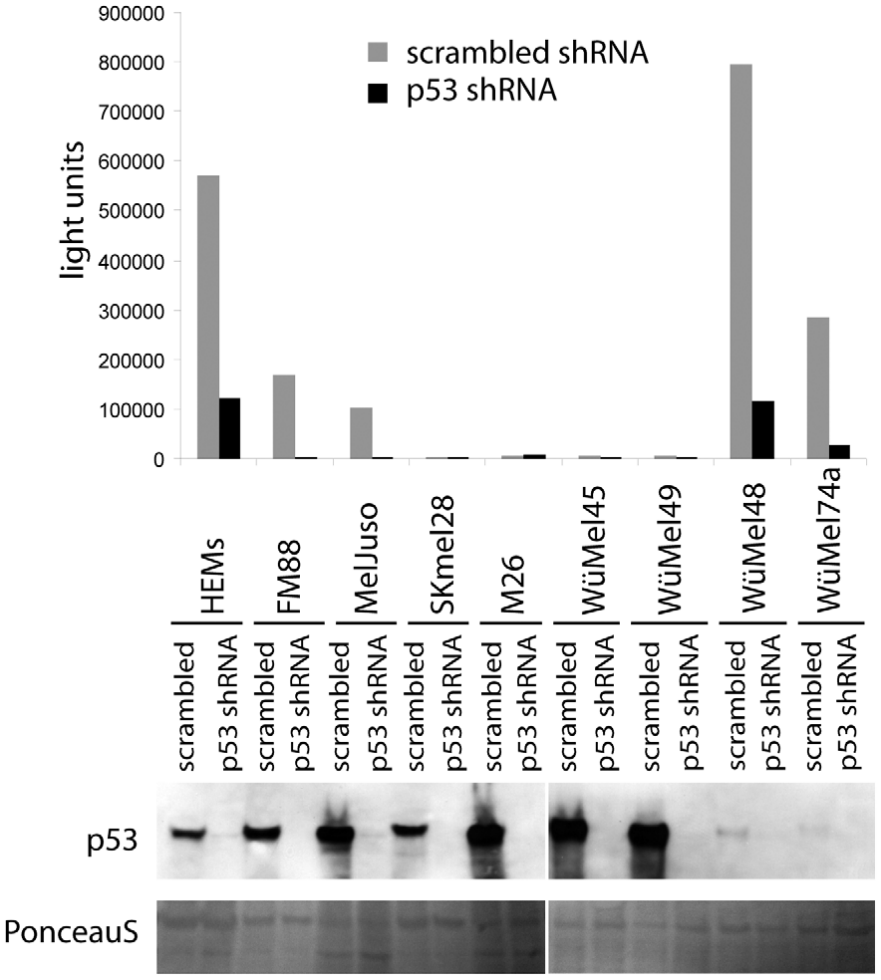
Functional inactivity in 4 out of 8 melanoma cell lines expressing wild type p53 measured by the p53 reporter construct pGreenFire®. Human epidermal melanocytes and the indicated melanoma cell lines were stably transduced with a lentiviral pGreenFire reporter construct encoding luciferase and GFP under the control of a p53 responsive element (4× CGACATGCCCGGGCATGT). The cells were then infected with lentiviral supernatants carrying the shRNA expression construct KH1 containing either a scrambled or a sequence targeting p53 [41]. On Day 4 following infection total cell lysates were analysed for p53 expression by immunoblotting (lower part). Equal loading was controlled by Ponceau S staining of the blot. In the upper part the corresponding luciferase activity is depicted, normalized to the relative luciferase load of the cell lines determined by Real time PCR.

Most of the above-described melanoma cell lines express high levels of p53 which are comparable to those found in melanoma cells *in situ*. To understand why melanoma cells tolerate high levels of wild type p53 expression we addressed the pertinent question whether p53 is transcriptionally active in these cells. To this end, in a first set of experiments 10 of the melanoma cell lines described above were stably transfected with a PathDetect® reporter plasmid coding for luciferase under the control of a p53 enhancer element. As expected, the 4 melanoma cell lines harbouring inactivating p53 mutations did not show p53-dependent luciferase expression (Figure 3). Surprisingly, however, among the 6 cell lines with wild type p53 p53-dependent luciferase expression which could be abolished upon shRNA knock down of p53 was only observed in FM88 and MelJuso cells (Figure 3). The presence of the luciferase DNA in the genome was confirmed by real time PCR for all 10 cell lines (in Figure 3 the relative copy numbers are given on top of the figure) and the functionality of the luciferase reporter could be demonstrated in 8 of the cell lines as luciferase expression was inducible upon transient ectopic expression of p53 (data not shown). Thus, in 4 out of 6 cell lines which reflect the *in situ* p53 status, no p53-dependent luciferase expression could be detected (Figure 3). Since it is known that different p53 responsive elements are targeted differentially by p53 depending on the cellular and environmental background [23] we tested a second reporter with a different p53 response element. As we, furthermore, aimed to analyze primary melanocytes as an untransformed control we used the lentiviral pGreenFire p53 reporter, which allowed efficient transduction of melanocytes. In addition to four of the p53 wild type melanoma cell lines analyzed by the PathDetect reporter in the first series of experiments, human epidermal melanocytes (HEM) as well as four short term melanoma cell lines carrying wild type p53 (WüMel) were analyzed in this set of experiments. The results obtained with the PathDetect p53 reporter were definitely reproduced with the pGreenFire p53 reporter for FM88, MelJuso, Skmel28 and M26 (Figure 4). Out of the four WüMel cell lines WüMel48 and 74a - which were proliferating only very slowly *in vitro* - displayed high, p53 shRNA repressible, p53 reporter gene activity, although p53 was expressed at relatively low levels (Figure 4). In contrast, WüMel45 and 49 were characterized by lack of p53 transcriptional activity in the presence of high levels of wild type p53 (Figure 4). A high level p53 transcriptional acitivity - almost comparable to those observed in the melanoma cells - measured in HEMs suggests that tolerating such p53 activities may be an intrinsic feature of melanocytic cells.

**Figure 5:**
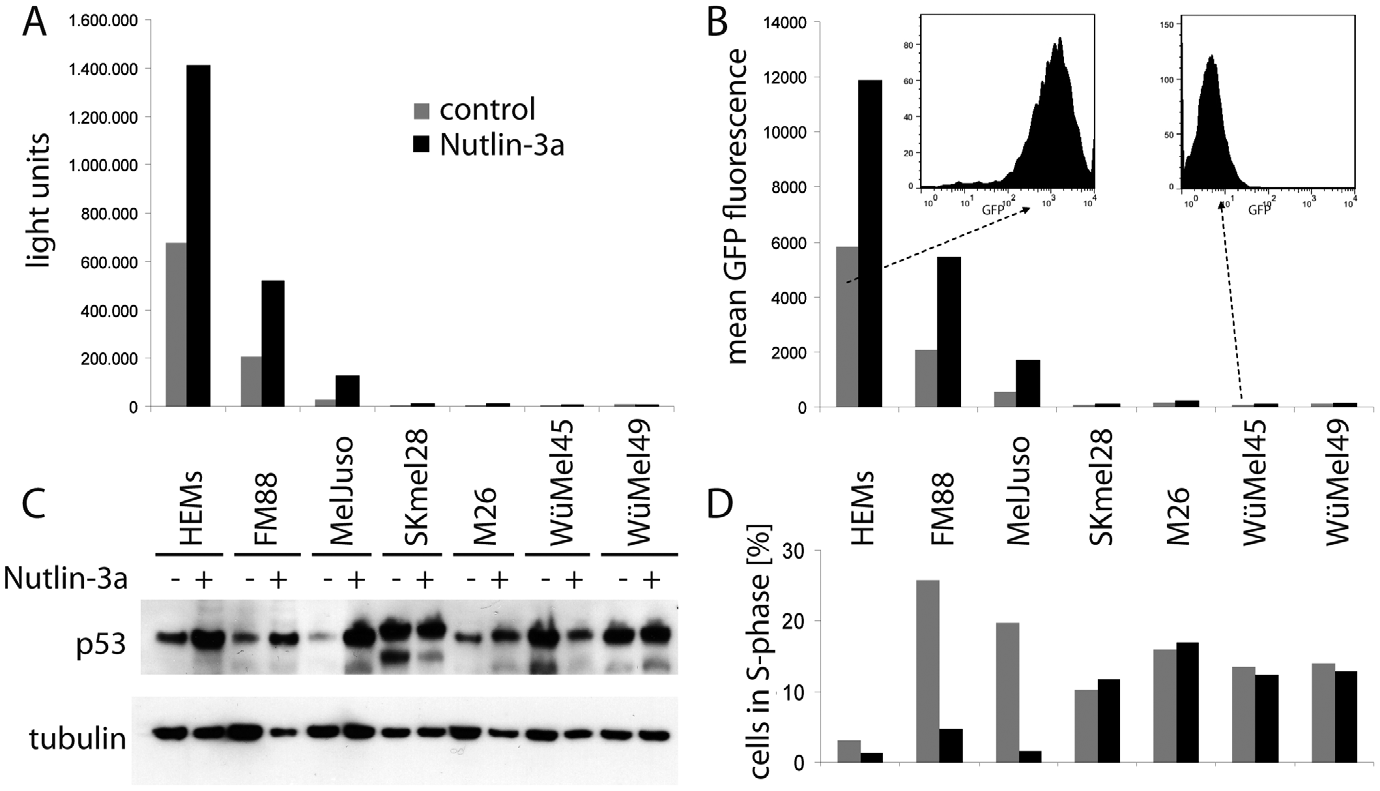
Determination of p53 reporter activities associated with cell cycle arrest. The indicated cells stably transduced with the pGreenFire reporter construct encoding luciferase and GFP under the control of a p53 reporter were cultured in the absence or presence of the mdm-2 inhibitor Nutlin 3a. After 24 hours p53 expression (C), luciferase acitvity (A) and GFP fluorescence (B) were analyzed. Cell cycle analysis was performed on day 2 of treatment and the percentage of cells in S-phase are depicted (D).

The pGreenFire vector contains luciferase and GFP which are both expressed under the control of a p53 responsive element. Results obtained for GFP fluorescence analyzed by flow cytometry (mean fluorescence intensity) and luciferase assays were highly concordant, indeed (Figure 5 A and B). Moreover, as flow cytometry allows analysis on a single cell level, we could exclude that high reporter gene activity in melanocytes or in the respective melanoma cell lines represents an average of high and low p53 activity in different sub-populations (Figure 5 B).

In order to rank the levels of p53 dependent luciferase/GFP expression with regard to the growth-inhibitory function of p53, we treated the cells with Nutlin-3a, a specific inhibitor of the p53 ubiquitinase mdm-2. This intervention induced both moderate increases in p53 expression (Figure 5 C) and p53 transcriptional activity (Figure 5 A and B); this moderate increase, however, was associated with a clear cut cell cycle arrest for HEMs, FM88 and MelJuso (Figure 5D). In contrast S-phase entry was not impaired in the low level p53 activity melanoma cell lines (Figure 5 D). In this context, SKmel28, WüMel45 and WüMel49 did not show an increase in p53 expression suggesting that mdm-2 does not restrict p53 expression in these cell lines (Figure 5C).

In summary our data demonstrate that p53 mutations are at best a rare event in melanoma *in situ*; still, strong protein expression in melanoma tissue indicates aberrations of p53 activity. Accordingly, we demonstrate functional inactivation of p53 in a considerable proportion (60%) of melanoma cell lines characterized by wild type p53. This notion is corroborated by a recent report of a gene expression profiling study which demonstrates that p53 target genes are differentially expressed in melanomas compared with nevi, strongly suggesting a dysfunctional p53 [24]. Moreover, our results are consistent with the reported UV response of melanoma cell lines [25]; i.e. despite p53 target gene induction, p53 DNA-binding activity was absent in melanoma cells with wild type p53. Interestingly, the authors further reported that p53 target genes were similarly induced in cells with mutant p53, thereby suggesting a comparable functional activity of the DNA damage pathway upon UV-irradiation in melanoma cells with wild type and mutated p53.

Future studies will be required to determine the mechanisms which lead to transcriptional inactivity of wild type p53 in melanomas. Possibly it involves proteins known to inhibit p53 like iASPP, ΔNp73, YB-1 or Parc through direct interaction [11]–[13], [26]. Alternatively, silencing might be achieved by posttranslational modifications. In this respect phosphorylation, acetylation, methylation, sumoylation and neddylation have been identified to impact p53 transcriptional activity [27]. Especially acetylation has been shown to alter p53 target specificity [28]. Recently Satyamoorthy and colleques reported that impairment in DNA damage induced phosphorylation of p53 on serine-376 may contribute to radioresistence of melanoma cells [29]. Moreover, the preservation and increase of wild type p53 expression during melanoma progression may be indicative of a tumor promoting function of functionally silenced wild type p53. Therefore, cytoplasmic functions of transcriptionally inactive p53 should be analysed since they may either limit or promote tumor growth [30], [31].

## Materials and Methods

### Ethics Statement

Written informed consent was obtained from all patients prior to any of these measures and the study was performed in adherence to the Declaration of Helsinki Principles. The medical ethical committee of the Universitätsklinik Würzburg approved all described studies. Generation of melanoma cell lines after written consent from the patients was approved by the Institutional Review Board of Würzburg University Hospital (Ethikkommission der Medizinischen Fakultät der Universität Würzburg; sequential study number 123/08_ff).

#### Tumor material and p53 sequencing

Paraffin-embedded tumor samples from primary and metastatic melanomas were obtained by surgical excision. All tumors had undergone routine histology for diagnosis. The three immediately following slides from the blocks were used for DNA extraction and the following slides were used to confirm the presence of the lesion and for immunohistochemistry.

### Sequence analysis of p53 exons 5 to 8

Prior to DNA extraction adjacent normal tissue was macroscopically dissected. For sequencing (SeqLab; Göttingen, Germany) the *p53* exons 5, 6 7 and 8 were amplified with BioTherm Taq polymerase (GenCraft, Münster, Germany) by semi-nested PCR using the following primers which all target exon flanking intron sequences:

Exon 5-1 GTGCCCTGACTTTCAACTCTG

Exon 5-2 ATCAGTGAGGAATCAGAGGC

Exon 5-3 (nested Primer) GGGCAACCAGCCCTGTCG

Exon 6-1 GCCTCTGATTCCTCACTGAT

Exon 6-2 GGAGGGCCACTGACAACCA

Exon 6-3 (nested Primer) CCAGAGACCCCAGTTGCAAAC

Exon 7-1 AGGCGCACTGGCCTCATCTT

Exon 7-2 AGGGGTCAGAGGCAAGCAGA

Exon 7-3 (nested Primer) TCAGAGGCAAGCAGAGGCTG

Exon 8-1 GGACAGGTAGGACCTGATTTCCTTAC

Exon 8-2 TGAATCTGAGGCATAACTGC

Exon 8-3 (nested Primer) TGCACCCTTGGTCTCCTCCAC

Sequences found to be affected by mutations were deposited at Genbank.

#### Immunohistochemistry

4 µm sections of paraffin-embedded tumors were dried at 56°C and then treated twice with xylene for 10 min at room temperature. Subsequently, sections were washed twice with absolute ethanol and twice with 70% ethanol followed by one rinse with bi-distilled water. For antigen retrieval, sections were incubated with citrate buffer pH 9.0 (DAKO, Hamburg, Germany) for 10 min at 90°C and rinsed with bi-distilled water. Next, slides were rinsed twice with phosphate-buffered saline (PBS) and thereafter incubated with Blocking Solution (DAKO, S2023) for 10 min at room temperature. After two additional washing steps with PBS for 10 min at room temperature, the monoclonal α-p53 antibody (DO-7, DAKO) was added to the sections in at a predetermined concentration in PBS, followed by an over night incubation at 4°C. After two 10 min washes in PBS, biotinylated multispecies-specific secondary antibody (DAKO, K5003) was added to the sections for 30 min at room temperature. Slides were then washed twice in PBS/bovine serum albumin, and bound antibodies were visualized using streptavidin-HRP (DAKO K5003) and Vector Vip (Vector Laboratories, Burlingame USA) as peroxidase substrate according to the manufacturer's guidelines. Finally, the nuclei were stained with hemalaun.

### Cell culture

Four melanoma cell lines (WüMel45, WüMel48, WüMel49 and WüMel74a) were generated in our lab from melanoma metastasis obtained from patients for diagnostic purposes, These cell lines as well as the melanoma cell lines BLM [32] FM88 [33] IF-6 [34], MV-3 [34] Mel2a [35] MelJuso [36] Skmel28 [37] FM55 [38] M19 [39] and M26 [39] were grown in RPMI 1640 supplemented with 10% fetal calf serum. Human epidermal melanocytes (HEM) were purchased from PromoCell (Heidelberg, Germany) and cultured in HAM's F10 media, supplemented with 20% fetal bovine serum, glutamine, ITS premix, 12-O-tetradecanoylphorbol-13-acetate, IBMX, and cholera toxin [40].

### Transfection and infection

To establish cells with an integrated p53 reporter plasmid the PathDetect® p53 *cis*-reporter gene construct (Strategene, La Jolla, CA, USA), which codes for luciferase under the control of a p53 responsive element (14× TGCCTGGACTTGCCTGG) as well as a puromycin resistance plasmid (pBabe-puro) were co-transfected in a ratio of 10/1. Then puromycin resistant cells were selected. Integration of the luciferase gene was confirmed by PCR. The second p53 reporter construct was the pGreenFire lentiviral vector (SBI, Mountain View, Canada) which codes for a puromycin resistence and for green fluorescent protein (GFP) as well as luciferase under the control of a different p53 responsive element (4× CGACATGCCCGGGCATGT). Knock down of p53 protein expression was achieved by a lentiviral shRNA expression construct KH1 containing either a scrambled or a sequence targeting p53 [41]. Infectious viruses were raised by transfecting HEK293T [42] cells with the pGreenFire construct or the respective KH1 vector and the helper constructs p59, p60 and p61. Two days following transfection, virus supernatants were harvested and filtered through 0.45 µm pore size filters. For infection, virus-containing supernatants were supplemented with 4 µg/ml polybrene and then added to the target cells overnight. Then medium was changed and the cells were cultured for 3 more days prior to subjecting lysates of the cells to further analysis. Pure populations carrying the pGreenFire reporter were selected by culturing the cells in the presence of puromycin.

### Real time PCR

To quantify the relative presence of the reporter constructs in the transfected or infected cells a real time PCR assay for luciferase was applied. Genomic DNA derived from the cell lines was analyzed using TaqMan technology with primers (forward: 5′- TTG GCA GAA GCT ATG AAA CG -3′; reverse: 5′-GCA ACT GCA ACT CCG ATA AA -3′) and a respective probe (TP: FAM-CGC CCA ACA CCG GCA TAA AGA -Tamra). By normalization to the highly repetitive DNA elements LINE1 - for which the copy number even in cancer cells is largely constant - a relative quantification of the samples could be calculated by the ΔΔ*C*_t_ method.

### p53 reporter gene assay

Following 2 washes with PBS, triplicates of infected cells were harvested by adding lysis buffer (10 mM NaCl, 8 mM tricine (ph 7.8), 0.4 mM EDTA, 1 mM DTT and 0.2%Triton X-100). After shaking for 20 min at room temperature, the lysates were frozen and stored at −20°C overnight. Upon thawing, the lysates were centrifuged and 50 µl of supernatant was transferred to a luminometer plate and luciferase acitivity was measured in an Orion II luminometer (Berthold, Pforzheim, Germany) following automatic injection of a luciferase buffer as previously described [43]. Flow cytometry to monitor GFP expression in pGreenFire transduced cells was performed on a FACSCanto (BD, Heidelberg, Germany).

### Western blot analysis

Cells were lysed in Laemmli buffer and proteins were resolved by SDS-polyacrylamid gel electrophoresis and transferred to nitrocellulose membranes. Following blocking for 1 h with PBS containing 0.05% Tween 20 and 5% powdered skim milk, blots were incubated overnight with the primary antibody, washed three times with PBS with 0.05% Tween 20, and then incubated with a peroxidase coupled secondary antibody. Following extensive washing, the bands were detected using a chemoluminescence detection kit (Roche Diagnostics, Mannheim, Germany). Antibodies to p53 (D-01) (Santa Cruz, Heidelberg, Germany) and β-tubulin (Sigma, Ottobrunn, Germany) were used.

### Cell cycle analysis

Cells were harvested and resuspended in 0.5 ml PBS supplemented with 1% fetal calf serum. Five ml of ice-cold EtOH was added, followed by an over night incubation at 4°C. The fixed cells were pelleted and resuspended in 1 ml PBS supplemented with 1% fetal calf serum, 0.05 mg/ml propidium iodide, and 0.1 mg/ml RNase A. Following a 1-hour incubation at 37°C, analysis of the cellular DNA content was performed on a FACSCanto flow cytometer.
